# RF-induced heating of capped and uncapped abandoned epicardial leads during MRI at 1.5 T and 3 T

**DOI:** 10.1109/EMBC40787.2023.10340533

**Published:** 2023-07

**Authors:** Fuchang Jiang, Kaylee R. Henry, Bhumi Bhusal, Gregory Webster, Giorgio Bonmassar, Daniel Kim, Laleh Golestanirad

**Affiliations:** Department of Biomedical Engineering, Northwestern University, Evanston, IL 60608 USA.; Department of Biomedical Engineering, Northwestern University, Evanston, IL 60608 USA.; Department of Radiology, Northwestern University, Chicago, IL 60611 USA.; Division of Cardiology, Ann and Robert H. Lurie Children’s Hospital of Chicago, Northwestern University Feinberg School of Medicine, 225 East Chicago Avenue, Box 21, Chicago, IL, 60611, USA; A. A. Martinos Center for Biomedical Imaging, Massachusetts General Hospital, Boston, MA, USA; Department of Radiology, Northwestern University, Chicago, IL 60611 USA.; Department of Radiology and Department of Biomedical Engineering, Northwestern University, Chicago, IL 60611 USA.

## Abstract

There is a paucity of data regarding the safety of magnetic resonance imaging (MRI) in patients with abandoned epicardial leads. Few studies have reported temperature rises up to 76 °C during MRI at 1.5 T in gel phantoms implanted with epicardial leads; however, lead trajectories used in these experiments were not *clinically* relevant. This work reports patient-specific RF heating of both capped and uncapped abandoned epicardial lead configurations during MRI at both 1.5 T and 3 T field strengths. We found that leads routed along realistic, patient-derived trajectories generated substantially lower RF heating than the previously reported worst-case phantom experiments. We also found that MRI at the head imaging landmark leads to substantially lower RF heating compared to MRI at the chest or abdomen landmarks at both 1.5 T and 3 T. Our results suggest that patients with abandoned epicardial leads may safely undergo MRI for head imaging, but caution is warranted during chest and abdominal imaging.

## Clinical Relevance—

Patients with abandoned epicardial leads may safely undergo MRI for head imaging, but caution is warranted during chest and abdominal imaging.

## I. Introduction

Magnetic resonance imaging (MRI) is a widely utilized imaging modality in modern medicine, however, its application in patients with cardiac implantable electronic devices (CIEDs) is limited due to the potential for heating of the tissue in the vicinity of implanted leads, known as the “antenna effect” [1–3]. This occurs as the result of the interaction between the electric field of the MRI scanner and the elongated wires of the implanted lead, which amplifies the specific absorption rate (SAR) of the radiofrequency (RF) energy in the surrounding tissue [4–6]. The safety of MRI in patients with abandoned epicardial leads, which are no longer connected to a pulse generator, poses a significant concern, as there is currently a lack of data on their MRI safety profile and no MR-conditional epicardial devices available [7]. One study that examined RF heating of epicardial leads reported alarmingly high temperature rises during MRI at 1.5 T [8], however, it did not consider realistic lead trajectory configurations, which are particularly important for abandoned epicardial leads due to their varying trajectory compared to endocardial leads.

When leads are abandoned without a pulse generator, surgeons must choose whether to place a plastic cap on the lead tip or to place the abandoned lead back uncapped in the pocket. That decision is driven by theoretical concerns of infection, pocket dimensions, and surgeon preference. This paper examines RF heating of both capped and uncapped abandoned epicardial leads during MRI at both 1.5 T and 3 T field strengths, and at different imaging landmarks. The study aims to determine how lead termination, MRI field strength and imaging landmark impact on the RF heating of such leads. The findings of this study will provide valuable information for patient safety and management of abandoned epicardial leads during MRI.

## II. Methods

### A. Phantom Design and Construction

**Figure 1.**
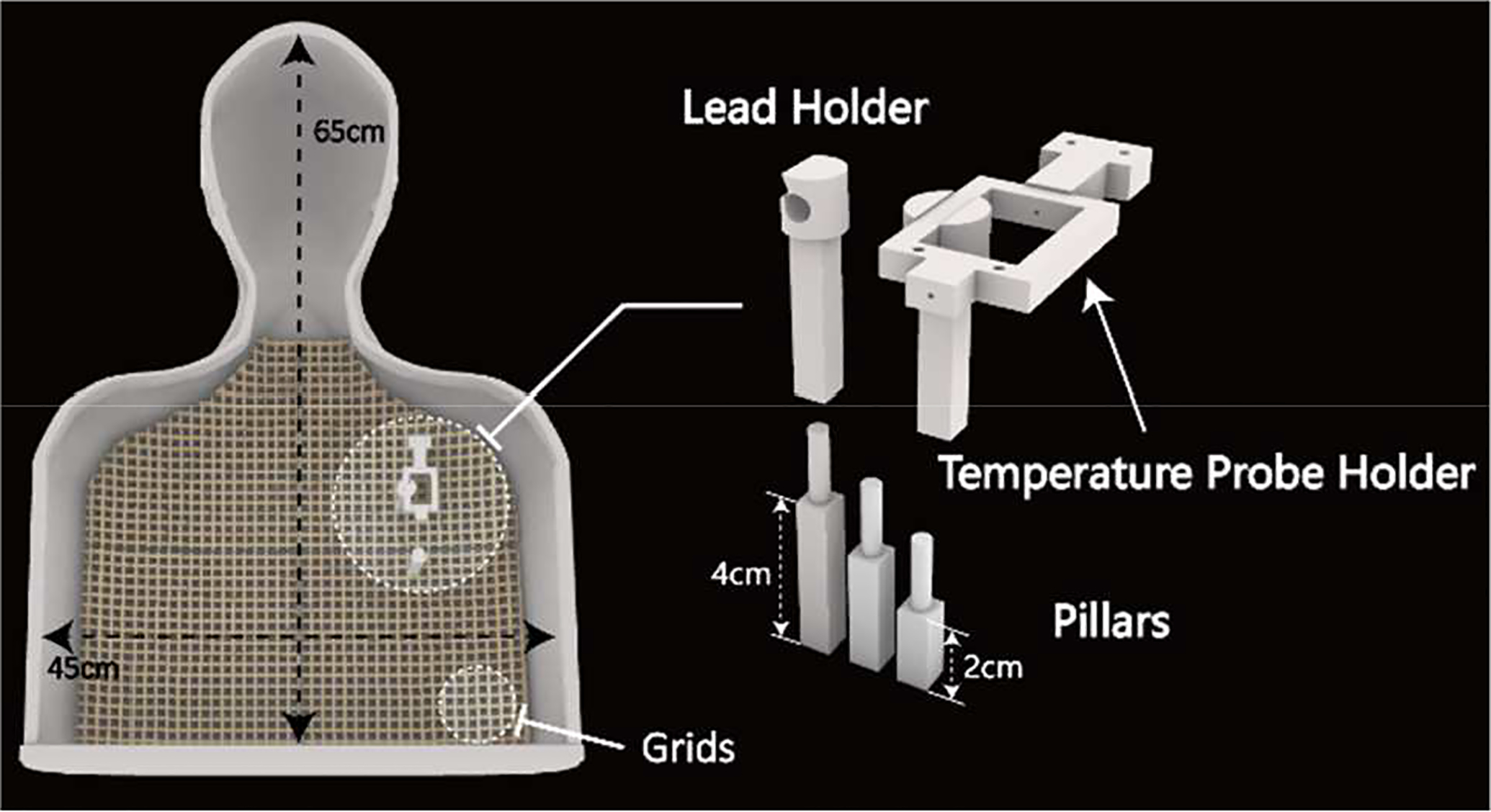
Left: Phantom (height=65 cm, width=45 cm, depth=14 cm) with grids and pillars. Right: Zoomed-in view of the custom-designed temperature probe holder, and pillars of varying heights used to adjust the positioning of the leads.

We performed *in-vitro* experiments with a custom-made, human-shaped phantom created from computed tomography (CT) images of an average-sized, middle-aged adult patient. Details of our image segmentation, surface model construction, and phantom fabrication are given elsewhere [9]. We also designed and 3D printed grids, pillars of varying heights, and lead holders to allow us reliably replicate clinically relevant lead configurations (Fig. 1). The phantom was filled with polyacrylamide (PAA) gel (22 L) consisting of gelled saline prepared by mixing 8g/L polyacrylamide (PAA) and 1.55 g/L NaCl in distilled water. The gel had a conductivity of σ= 0.47 S/m and a relative permittivity of ε_r_= 88 at 64 MHz as measured by using a vector network analyzer (Keysight Technologies, Santa Rosa, CA) along with a dielectric measurement kit (N1501A). These dielectric properties were chosen as they result in a tissue-mimicking medium. Leads were placed ~2 cm below the surface of the gel similar to the depth at which they are implanted in patients.

### B. Epicardial Lead Configurations

**Figure 2.**
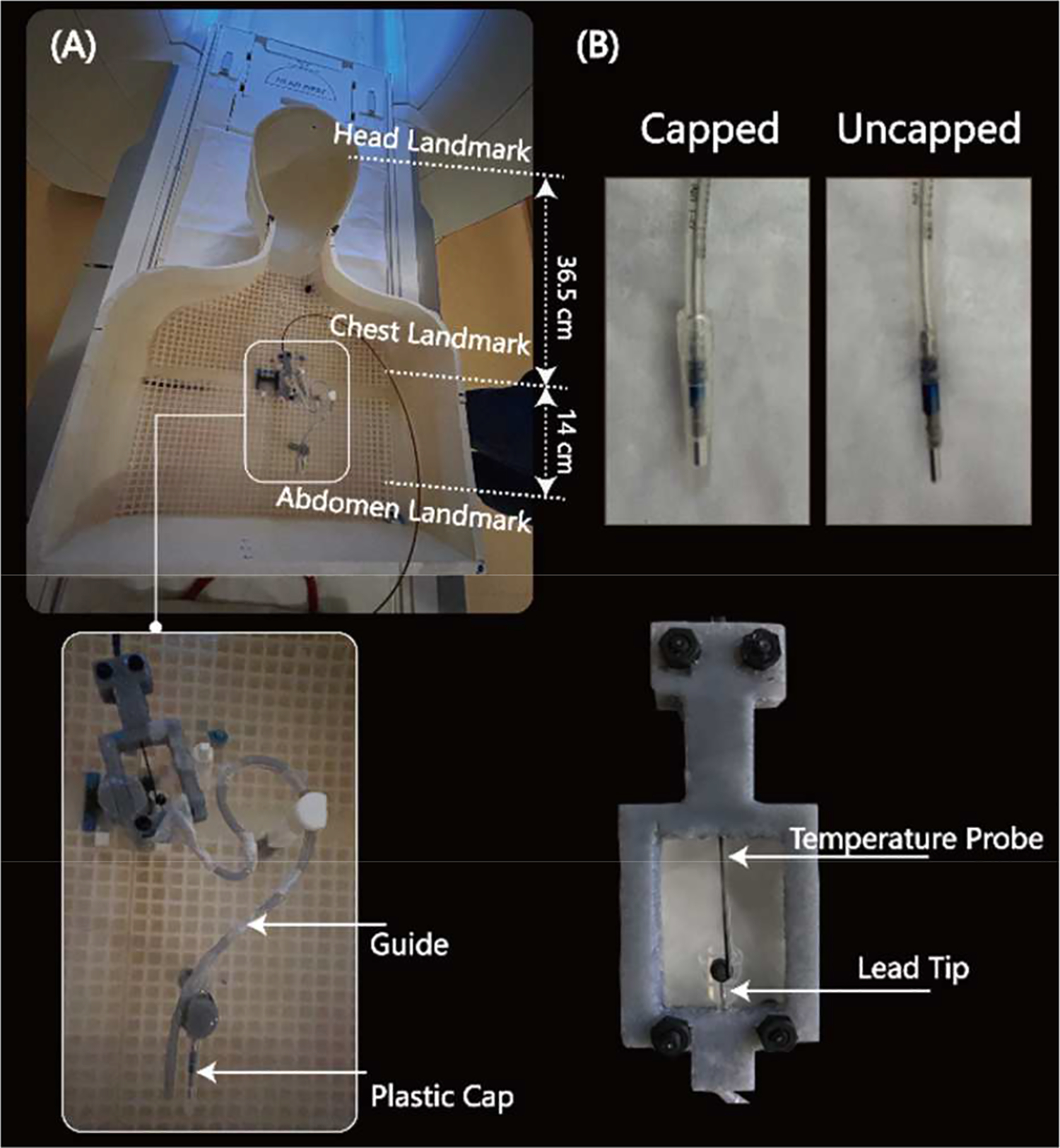
(A) Phantom with lead and temperature measurement setup. (B) Closer view illustrating the contact between the temperature probe and epicardial lead tip.

**Figure 3.**
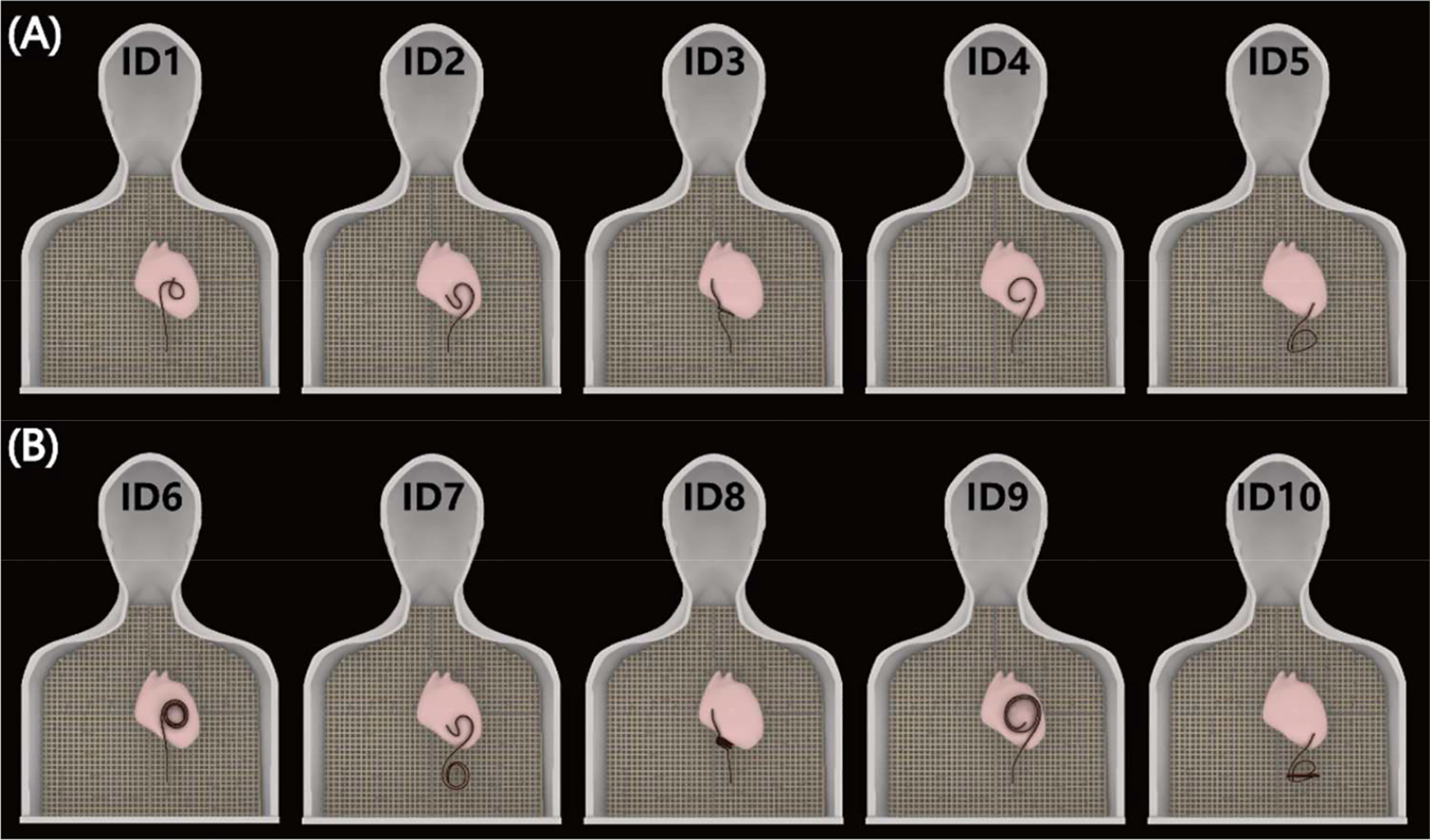
Ten distinct clinically relevant epicardial lead trajectories were investigated, including (A) five 25 cm leads and (B) five 50 cm leads.

It is well established that the trajectory and orientation of an elongated implant with respect to the MRI electric field substantially affects its RF heating [10–18]. For this reason, we replicated 10 clinically relevant lead configurations derived from patients’ CT or X-Ray images or designed based on the expert opinion of a pediatric electrophysiologist. Two commercially available epicardial leads (Medtronic CapSure^®^ EPI 4965) at lengths of 25 cm and 50 cm were used in experiments, each tested at N=5 unique trajectories. The proximal end of the lead was either capped or exposed to the gel, depending on the experimentation configuration being tested. To allow for stable and reproducible placement of the leads, we 3D printed plastic guides which helped in routing the leads along patient-specific trajectories and kept the leads securely in place during the experiments (Fig. 2A). The location of the lead’s proximal end was fixated at ~15 cm caudal to the center of the heart for all trajectories (Fig. 3).

### C. RF Exposure

Temperature measurements were performed using MR-compatible fiber optic probes (OSENSA, Vancouver BC, Canada, resolution 0.01°C) secured at the tip of the lead. To ensure reliable thermal contact, we 3D printed a custom-designed holder that securely held the temperature probe in place so that it was in direct contact with the tip of the lead throughout the experiment (Fig. 2B).

RF exposure was performed in a 1.5 T Siemens Aera scanner and a 3T Siemens Prisma scanner (Siemens Healthineers, Erlangen, Germany). The phantom was placed inside the scanner in the head-first, supine position and experiments were performed at various landmarks corresponding to head, chest, and abdomen imaging. The phantom was registered as a patient with a height of 5 feet 5 inches (165 cm) and a weight of 150lb (68kg).

A high-SAR steady-state free precession (SSFP) sequence (TE = 1.69 ms, TR = 3.44 ms, Acquisition time = 315 s) and a T_1_-weighted turbo spin echo (T_1_-TSE) sequence (TE = 7.5 ms, TR = 1450 ms, Acquisition time= 451 s) were used for the temperature measurements at 1.5 T and 3 T, respectively. Flip angles were adjusted in each configuration to reach the maximum SAR limit of the scanners.

## III. Results

**Figure 4.**
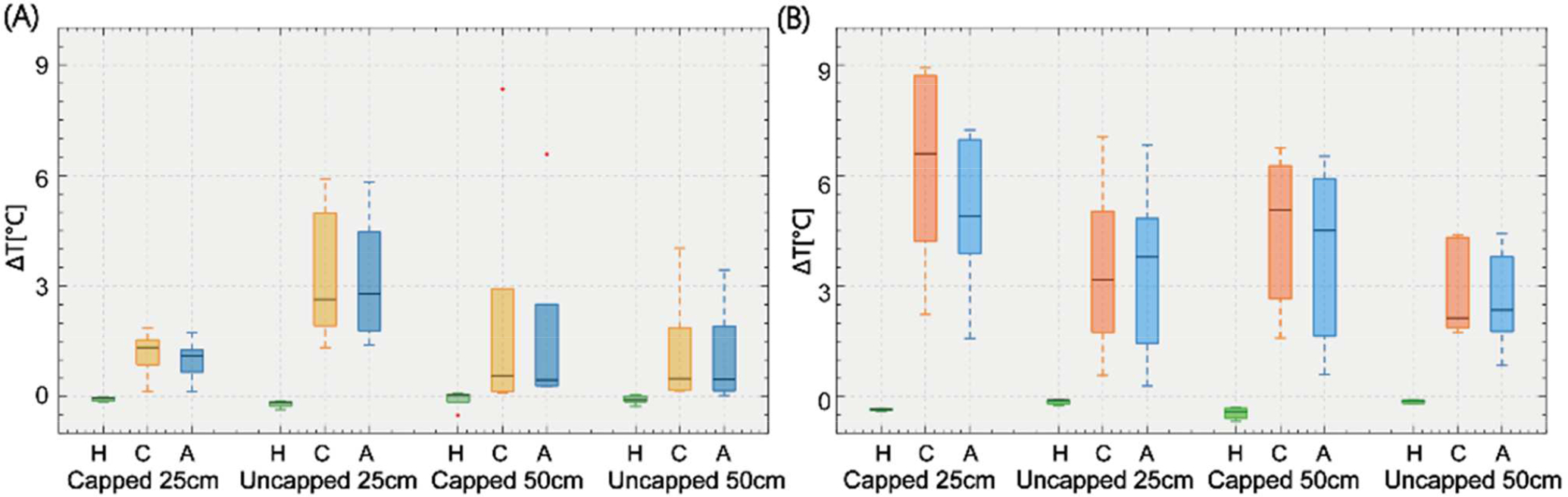
Temperature rise at lead tip at various landmark positions, with variations in lead length and capped/uncapped. (A) 1.5 T and (B) 3T. Note: H = Head landmark, C = Chest landmark, A = Abdomen landmark

Table 1 reports the values of the measured temperature rise and scanner-reported rms B_1_^+^ for each trajectory at 1.5 T and 3 T. For each experiment, the flip angle (and by proxy, B_1_^+^) was adjusted to generate the maximum allowable SAR. Figure 4 illustrates the temperature rise distributions for each experimental group. Data normality was tested with a Shapiro-Wilk’s test for each group. An unpaired two sample t-test was used to compare the means when both groups of data were normally distributed. An unpaired two-sample Mann Whitney Wilcoxon test was used to compare the medians when at least one group of data was not normally distributed.

### A. RF Heating at 1.5 T vs. 3 T

At 1.5 T, the mean ± standard deviation of RF heating was −0.10 ± 0.15°C when the phantom was positioned at the head imaging landmark, 1.94 ± 2.20°C at the chest imaging landmark, and 1.76 ± 1.88°C at the abdomen imaging landmark (data pooled over both lead lengths and termination configurations). At 3 T, the mean ± standard deviation of RF heating was −0.24 ± 0.21°C for the head imaging landmark, 4.28 ± 2.44 °C for the chest landmark, and 3.74 ± 2.21°C for the abdomen landmark (pooled over lead lengths and termination configurations). Most of our reported temperature rises measured at the head imaging landmark were negative values, which indicated the gel was slowly cooling down during the experiment as the induced power was low.

A one-tailed Mann Whitney Wilcoxon test at significance level of α = 0.05 revealed that the heating generated at 3 T was significantly higher than at 1.5 T for capped leads at both the chest and abdomen imaging landmarks (both p<0.005). However, there was no significant difference in the heating of the uncapped leads (p=0.97 for head; p= 0.24 for chest; p= 0.32 for abdomen).

### B. Effect of Imaging Landmark

RF heating of epicardial leads was significantly lower at the head imaging landmark compared to the chest landmark (p<0.005 for both 1.5T and 3 T) and abdomen landmarks (p<0.005 for both 1.5T and 3 T). An F-test and Levene’s test were used to test the homogeneity of the variance for our normally distributed and non-normally distributed data, respectively. There was less variability in the RF heating measurements at the head landmark, compared to the chest and abdomen (p<0.005 for both 1.5T and 3T). There was no significant difference between RF heating at the chest and abdomen landmarks (p=0.9 at 1.5 T; p=0.5 at 3T).

### C. Capped vs. Uncapped Leads

The effect of lead termination depended on lead’s length, MRI field strength, and imaging landmark.

For the 25 cm lead at 1.5 T, uncapped termination led to a significantly higher RF heating compared to the capped termination (1.08±0.58°C vs 3.26±1.73°C, p=0.002) for chest and abdomen landmarks. The trend was, however, reversed at 3 T where capped 25 cm leads generated significantly higher heating at chest and abdomen landmarks (5.65±2.47°C vs 3.42±2.32°C, p=0.03). For the 50 cm lead, there were no significant differences between RF heating of capped and uncapped leads at 1.5 T (p=0.82) nor 3 T (p=0.10).

## IV. Discussion

Epicardial pacing is often a life-saving treatment in infants and young children with congenital heart diseases [19, 20]. Due to a child’s smaller anatomy and limited access to the chambers of the heart, the common implantation practice is to affix the epicardial lead directly to the myocardium (as opposed to endocardial leads in adults, that are passed through the subclavian vein) and place the implantable pulse generator (IPG) inferior to the abdominal rectus (as opposed to placing it in a subpectoral pocket). Because there is no straightforward method to extract epicardial leads, they are often disconnected from the IPG and left *in situ* when the patient no longer needs the device, or when an endocardial CIED is implanted later in life as the patient grows older. Caps are radio-lucent; therefore, it can be challenging for clinicians considering MR imaging months or years later to determine whether a lead is capped or uncapped. Therefore, it is critical to understand lead heading performance in the capped and uncapped status to allow for patient-specific estimations of average-case and worst-case heating scenarios. Because epicardial leads follow a substantially different trajectory than endocardial leads, their RF heating profile during MRI is also intrinsically different, warranting studies with clinically relevant trajectories [21, 22].

The data on MRI-induced RF heating of epicardial leads is scarce. One recent *in-vitro* study reported alarmingly high levels of temperature rise in a gel phantom (up to ~76 °C) during MRI at 1.5 T [8]. In that study however, the high heating was seen only for leads positioned close to the phantom walls and following a straight trajectory. Other studies confirm that the worst-case scenario heating is due to straight lead trajectories or placement near the edge of the phantom as well [13, 23]. In a realistic scenario, leads of implanted active electronic medical devices are usually looped at different locations to accommodate for the excess length (i.e., around the IPG, or in the case of neuromodulation devices such as deep brain stimulation devices, on the surface of the skull [24, 25]). Introduction of these loops has shown to reduce RF heating of deep brain stimulation leads at their tip [25, 26]. Similarly, epicardial leads are typically looped around the IPG or on the surface of the heart. The maximum RF heating that we observed in our clinically relevant configurations was between 8 and 9°C, which was ~ 9 fold less than the worst-case scenario reported for a straight lead in [8].

In this study we found RF heating to be higher at 3 T compared to 1.5 T for capped leads (1.49±2.13 vs 4.92±2.41, p<0.005) at the chest and abdomen landmarks, but not for uncapped leads. This agrees with previous works that concluded higher MRI field strengths do not necessarily generate higher RF heating around tips of implanted leads [27, 28].

Most importantly, we found that the choice of imaging landmark had a substantial effect on RF heating at both 1.5 T and 3 T field strengths. Specifically, RF heating remained below 0.09°C for all cases when the phantom was positioned with its head at the isocenter and the variability surrounding that measurement was small. This is clinically important because head imaging remains the most common indication to perform an emergent MRI, when there may be insufficient time to determine the details of a pacing system beyond inspection of a chest radiograph.

It should be pointed out, however, that although our study showed that epicardial leads with realistic trajectories generated lower RF heating than extreme values reported in the literature for worst-case scenarios, caution is still warranted when scanning patients at chest or abdomen landmarks. For a 15-minute scan, a 9°C temperature rise will be equivalent to a cumulative thermal dose of CEM43°C=120 minutes, close to levels that caused necrosis in pig muscles [29].

## V. Conclusion

Our results indicate that the MRI-induced RF heating around the tip of an abandoned epicardial lead could be substantially lower in patients compared to values reported in the literature for the worst-case scenario. Specifically, our results suggest that patients with abandoned epicardial leads are at low risk when undergoing MRI at both 1.5 T and 3 T for head imaging. This agrees with retrospective studies that reported no adverse effects in patients with abandoned leads undergoing MRI [30, 31]. While the majority of lead configurations demonstrated minimal, subclinical heating during chest and abdomen imaging, our results also call for caution. Some lead configurations may be associated with higher temperature elevations during chest and abdominal imaging.

## Figures and Tables

**Table 1:** The (RMS B_1_^+^ values /measured temperature rises) for each trajectory shown in Figure 3. LM =Landmark (e.g. CH = capped lead with head landmark; UH = uncapped lead with head landmark).

	ID1		ID2		ID3		ID4	
LM	1.5T	3T	1.5T	3T	1.5T	3T	1.5T	3T
**CH**	3.8μT/−0.03	2.3μT/−0.32	3.8μT/−0.04	2.3μT/−0.34	3.8μT/−0.01	2.4μT/−0.39	3.8μT/−0.14	2.3μT/−0.36
**CC**	4.9μT/0.15	2.8μT/4.87	4.9μT/1.10	2.8μT/6.59	4.9μT/1.43	2.8μT/8.92	4.8μT/1.33	2.8μT/8.64
**CA**	4.8μT/0.13	2.8μT/4.90	4.9μT/0.84	2.8μT/4.65	4.9μT/1.11	2.8μT/6.88	4.9μT/1.10	2.8μT/7.23
**UH**	3.8μT/−0.35	2.3μT/−0.09	3.8μT/−0.15	2.3μT/−0.10	3.8μT/−0.11	2.4μT/−0.18	3.8μT/−0.22	2.3μT/−0.10
**UC**	4.9μT/2.64	2.8μT/3.17	4.9μT/4.67	2.8μT/4.36	4.9μT/2.12	2.8μT/2.13	4.9μT/5.90	2.8μT/7.05
**UA**	4.8μT/2.79	2.8μT/3.79	4.9μT/4.02	2.8μT/4.17	4.9μT/1.91	2.8μT/1.83	4.9μT/5.82	2.8μT/6.82
	ID5		ID6		ID7		ID8	
LM	1.5T	3T	1.5T	3T	1.5T	3T	1.5T	3T
**CH**	3.8μT/−0.11	2.4μT/−0.35	4.5μT/0.06	2.4μT/−0.56	3.8μT/0.09	2.3μT/−0.32	3.8μT/0.04	2.4μT/−0.29
**CC**	4.9μT/1.86	2.8μT/2.23	4.9μT/1.12	2.8μT/5.07	4.9μT/0.57	2.8μT/3.01	4.9μT/0.09	2.8μT/1.59
**CA**	4.9μT/1.74	2.8μT/1.58	4.8μT/1.15	2.8μT/5.71	4.9μT/0.45	2.8μT/1.99	4.9μT/0.29	2.8μT/0.60
**UH**	3.8μT/−0.16	2.8μT/−0.24	4.5μT/−0.10	2.4μT/−0.19	3.8μT/0	2.3μT/−0.10	3.8μT/0.04	2.4μT/−0.11
**UC**	4.9μT/1.33	2.8μT/0.58	4.9μT/1.14	2.8μT/4.38	4.9μT/0.17	2.8μT/1.75	4.9μT/0.16	2.8μT/2.13
**UA**	4.9μT/1.40	2.8μT/0.29	4.8μT/1.40	2.8μT/4.42	4.9μT/0.03	2.8μT/0.85	4.9μT/0.20	2.8μT/2.07
	**ID9**		**ID10**					
LM	1.5T	3T	1.5T	3T				
**CH**	4.1μT/−0.51	2.3μT/−0.41	4.0μT/−0.03	2.4μT/−0.67				
**CC**	4.7μT/8.34	2.8μT/6.74	4.8μT/0.16	2.8μT/6.10				
**CA**	4.9μT/6.58	2.8μT/4.51	4.9μT/0.28	2.8μT/6.52				
**UH**	4.1μT/−0.26	2.3μT/−0.09	4.0μT/−0.09	2.4μT/−0.18				
**UC**	4.8μT/4.03	2.8μT/4.30	4.9μT/0.49	2.8μT/1.91				
**UA**	4.9μT/3.44	2.8μT/3.59	4.9μT/0.47	2.8μT/2.35				
